# Infantile-Onset Vanishing White Matter Disease in an Azerbaijani Infant With a Homozygous EIF2B5 p.(Arg195His) Variant

**DOI:** 10.7759/cureus.103917

**Published:** 2026-02-19

**Authors:** Cavid Isayev, Ilaha Haciyeva, Emil Hasanov, Fidan Hasanova, Parviz Samadov

**Affiliations:** 1 Neuroradiology, Independent Consultancy, Baku, AZE; 2 Neurology, Children’s Neurological Hospital, Baku, AZE; 3 Radiology, Liv Bona Dea Hospital, Baku, AZE; 4 Neurology, Baku Health Center, Baku, AZE

**Keywords:** cach, cree leukoencephalopathy, eif2b5, infantile-onset seizures, leukodystrophy, vanishing white matter disease

## Abstract

An eight-month-old Azerbaijani male infant, born to consanguineous (first-cousin) parents, presented with developmental regression and daily seizures following a febrile illness. He achieved head control at six months (delayed). During the same month, a rotavirus infection (fever, vomiting, and diarrhea) precipitated focal and generalized seizures. Neurological examination at eight months demonstrated severe hypotonia, hyperreflexia, and markedly reduced voluntary movements, with preserved visual and auditory responses. Brain MRI showed diffuse supra- and infratentorial white matter T1 hypointensity and marked T2 hyperintensity, with loss of subcortical U-fibers and deep white matter fluid-attenuated inversion recovery hypointensity consistent with rarefaction/degeneration, while the basal ganglia and cerebral cortex were relatively spared, an imaging pattern highly suggestive of advanced vanishing white matter (VWM) disease. Genetic testing identified a homozygous *EIF2B5* c.584G>A (p.Arg195His) variant, supporting the diagnosis. This case illustrates subacute infantile VWM with stressor-related neurologic deterioration, hypotonia, and refractory seizures, underscoring the value of early molecular diagnosis in infants with suspected leukodystrophy.

## Introduction

Vanishing white matter (VWM) disease, also termed childhood ataxia with central hypomyelination (CACH), is a rare autosomal recessive leukodystrophy caused by biallelic pathogenic variants in any of the five genes encoding the subunits of eukaryotic translation initiation factor 2B (*EIF2B1*-*EIF2B5*) [1]. It is most commonly recognized in childhood and typically presents with progressive cerebellar ataxia, pyramidal signs/spasticity, and variable optic atrophy. VWM shows a broad phenotypic spectrum, ranging from severe prenatal and infantile-onset forms to early childhood-, late childhood/juvenile-, and adult-onset disease [1]. Across phenotypes, neurological decline is generally chronic and progressive but is characteristically punctuated by episodes of acute, often irreversible, deterioration triggered by physiological stressors, including febrile infections, minor head trauma, or surgical interventions. Febrile illness is among the most frequent precipitants [1,2].

At the molecular level, eIF2B is a multi-subunit protein complex that helps “switch on” protein production in cells at the start of mRNA translation. During stress, cells normally reduce overall protein synthesis and activate protective pathways (the integrated stress response) to restore homeostasis [1,3]. Pathogenic *EIF2B* variants reduce this adaptive capacity; as a result, brain support cells (particularly oligodendrocytes and astrocytes) are unusually vulnerable to common stressors, providing a biologic rationale for the distinctive stress-triggered stepwise decline and progressive white matter rarefaction seen on MRI.

From a practical standpoint, early recognition matters because diagnostic certainty changes management priorities: avoiding and promptly treating febrile illnesses, minimizing head trauma and other triggers, anticipatory counseling about the risk of rapid decompensation, and timely genetic counseling for families (including recurrence risk in future pregnancies). In infants, the presentation may be dominated by developmental delay, hypotonia evolving into pyramidal signs, and seizures.

An entity historically termed Cree leukoencephalopathy (CLE) is now recognized as a severe early-infantile subtype within the VWM spectrum and is strongly linked to the *EIF2B5* c.584G>A (p.Arg195His) variant described as a founder variant in the Cree population. There are limited data on this variant in Central Asian/Azerbaijani cohorts, making this case clinically and epidemiologically relevant.

We report an Azerbaijani infant born to consanguineous parents who developed infection-triggered motor regression with refractory seizures. MRI features were highly suggestive of advanced VWM, with molecular confirmation of a homozygous *EIF2B5* c.584G>A (p.Arg195His) variant. The specific aim of this report is to (i) provide a clear clinical-radiologic-genetic correlation associated with the *EIF2B5* p.Arg195His variant in severe infantile-onset VWM, (ii) emphasize the practical diagnostic clue of stressor-related decompensation in infants presenting with seizures and developmental delay, and (iii) broaden the geographic evidence base for this genotype-phenotype association to support earlier recognition and genetic counseling in similar clinical settings.

## Case presentation

An eight-month-old Azerbaijani male infant was born to healthy first-cousin parents. He was the third live-born child; his two older siblings were healthy, and a prior pregnancy ended in intrauterine fetal demise due to placental abruption (considered unrelated). Pregnancy was otherwise uncomplicated, and delivery was by cesarean section at 39 weeks’ gestation. Birth weight was 4.1 kg, with Apgar scores of 8 and 9 at one and five minutes, respectively. Head circumference at birth was 37 cm (>97th percentile for sex and gestational age). Early developmental delay was noted: head control was achieved at six months, and independent sitting had not yet been attained. There were no parental concerns regarding vision or hearing.

At six months of age, the infant developed febrile gastroenteritis due to rotavirus, characterized by fever (up to 38.5°C), vomiting, and diarrhea. On day three of illness, seizures began as focal motor events involving a single limb, with subsequent spread to other body regions. Despite antiseizure medication, seizure frequency increased to multiple episodes per day. Following the illness, the parents reported loss of previously acquired head control and sitting ability, together with a marked reduction in purposeful hand use, including diminished grasping and toy holding. Over the subsequent weeks, no developmental gains were observed, and the child appeared to continue to decline.

At eight months of age, neurological examination demonstrated marked axial hypotonia with diffusely brisk deep tendon reflexes. He lacked head control, was unable to sit independently, and showed no purposeful hand use. Cranial nerve examination was unremarkable: visual tracking and auditory responses were preserved. Appendicular ataxia could not be reliably assessed given his inability to sit unsupported. Fundoscopy revealed no evidence of optic atrophy. The infant was awake and alert between seizures; ictal awareness appeared variably impaired. Increased tone consistent with spasticity was noted in the lower limbs.

The patient continued to experience daily seizures, including generalized tonic-clonic seizures and clusters of myoclonic jerks, typically lasting several minutes. Inpatient treatment with valproate reduced, but did not abolish, seizure frequency. Following discussion of the treatment strategy and expected prognosis with the patient’s legal guardians, antiseizure therapy was initiated with clobazam in combination with lacosamide, with a further reduction in seizure burden, although complete seizure control was not achieved.

Two brief intensive care unit admissions occurred at seven and eight months of age for status epilepticus. During both admissions, no ventilatory support beyond supplemental oxygen was required, and there was no respiratory failure. During the hospitalization at seven months of age, a “mitochondrial cocktail” (coenzyme Q10, L-carnitine, and vitamins) was administered, and levetiracetam was initiated for presumed mitochondrial encephalopathy. This was associated with a transient clinical improvement, characterized by reduced seizure frequency and a slight increase in tone; however, this benefit waned after discharge.

Extensive metabolic evaluation during the acute phase, including blood lactate, ammonia, plasma amino acids, urine organic acids, and an acylcarnitine profile, was unremarkable. Routine full blood count and liver function tests were within reference ranges. Despite these interventions, the clinical course remained progressive, with ongoing neurological deterioration on follow-up, and the patient died approximately three months after clinical onset.

MRI findings

Brain MRI was performed on a 3T scanner, including T1-weighted (repetition time (TR)/echo time (TE) = 4,000/371 ms) and T2-weighted (TR/TE = 8,360/103 ms) imaging, fluid-attenuated inversion recovery (FLAIR) (TR/TE = 5,830/89 ms), and diffusion-weighted imaging (DWI) (b = 1,000 s/mm²) with the corresponding apparent diffusion coefficient maps.

**Figure 1:**
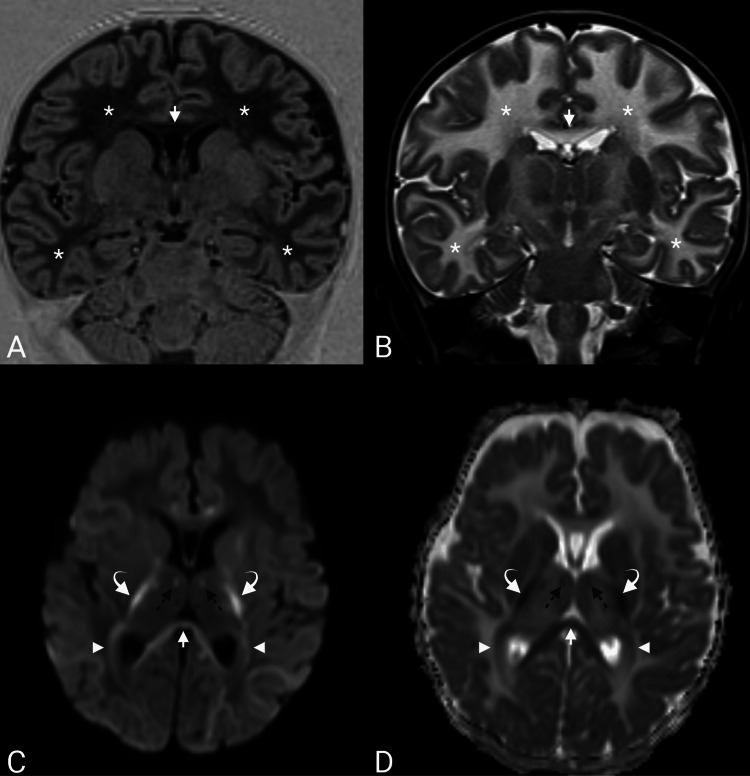
Diffuse dysmyelination with superimposed diffusion restriction. (A) Coronal T1 inversion recovery and (B) T2-weighted images show diffuse T1 hypointensity and T2 hyperintensity involving the deep and subcortical white matter (white asterisks) and the corpus callosum (white arrows). (C) Diffusion-weighted imaging and the corresponding (D) apparent diffusion coefficient map demonstrate restricted diffusion involving the internal capsules (curved white arrows), corpus callosum (white arrows), mammillothalamic tracts (black dotted arrows), and optic radiations (white arrowheads).

Brain MRI demonstrated diffuse, symmetric supratentorial white matter abnormality with T1 hypointensity and marked T2 hyperintensity, without appreciable sparing of the subcortical U-fibers (Figures 1A, 1B). At eight months of age, normal myelination is expected to include the corticospinal tracts, genu of the corpus callosum, and centrum semiovale, with ongoing progression in the anterior limb of the internal capsule. Therefore, the diffuse symmetric T2/FLAIR white matter abnormality observed in this patient exceeded age-appropriate maturation and was pathological [4]. DWI demonstrated multifocal areas of diffusion restriction involving the internal capsules, corpus callosum, mammillothalamic tracts, optic radiations, and dentate nuclei (Figures 1C, 1D). This pattern of restricted diffusion is nonspecific and may also be observed in instances of peri-ictal changes or hypoxic-ischemic injury. Considering the otherwise characteristic pattern of VWM and the clinical deterioration associated with stressors, superimposed stressor-related decompensation or intramyelinic edema was deemed a plausible unifying explanation.

**Figure 2:**
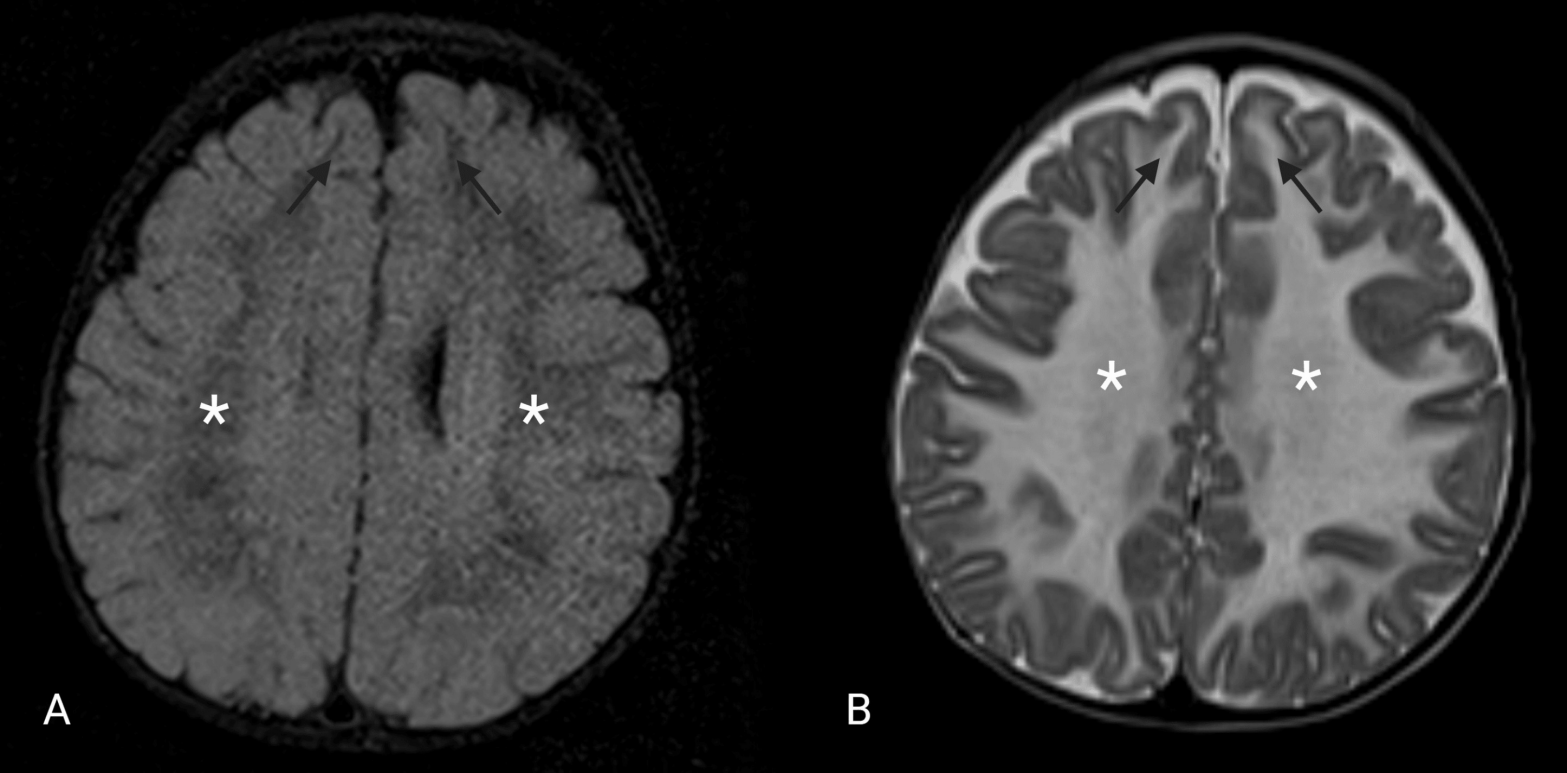
Supratentorial white matter rarefaction on T2 and FLAIR. (A) Axial fluid-attenuated inversion recovery (FLAIR) and (B) T2-weighted images at the frontoparietal level demonstrate diffuse, symmetric T2 hyperintensity involving the cerebral hemispheric white matter (white asterisks), including the subcortical white matter, without relative sparing of the subcortical U-fibers (black arrows). The deep white matter (white asterisks) shows corresponding marked FLAIR hypointensity. No definite signal abnormality is identified in the cerebral cortex.

The deep cerebral white matter was markedly hypointense on FLAIR, consistent with advanced rarefaction and degeneration (Figures 2A, 2B). Diffuse supratentorial white matter rarefaction provides a structural correlate for developmental regression and progressive loss of motor skills.

**Figure 3:**
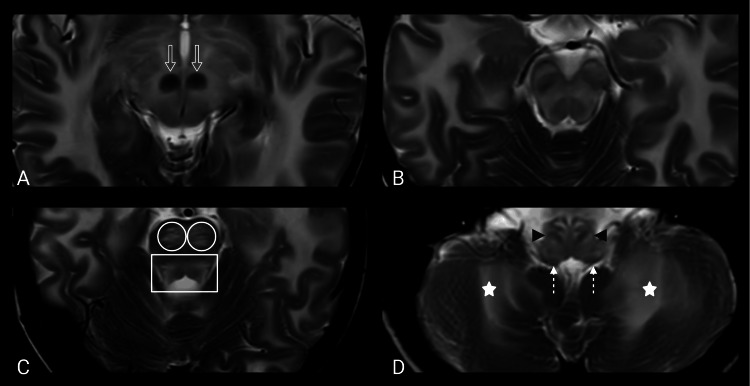
Brainstem and cerebellar involvement. (A, B) Midbrain level demonstrates T2 hyperintensity with relative sparing of the red nuclei (open white arrows). (C) At the upper pons, bilateral corticospinal tracts (white circles) and the pontine tegmentum (white rectangle) demonstrate T2 hyperintensity. (D) At the medulla, there is T2 hyperintensity with relative preservation of the inferior olivary nuclei (black arrowheads) and inferior cerebellar peduncles (white dotted arrows), and the deep cerebellar white matter (white stars) is symmetrically affected.

Infratentorial involvement was also observed. Diffuse midbrain T2 hyperintensity was observed; the red nuclei appeared relatively spared and were therefore more conspicuous against the surrounding hyperintense parenchyma (Figures 3A, 3B). At the pontine level, the bilateral corticospinal tracts and the pontine tegmentum demonstrated T2 hyperintensity (Figure 3C). Bilateral corticospinal tract involvement aligns with an anatomic correlate of evolving spasticity. The medulla oblongata also showed T2 hyperintensity with relative preservation of the inferior olivary nuclei and inferior cerebellar peduncles. The deep cerebellar white matter was symmetrically affected (Figure 3D).

**Figure 4:**
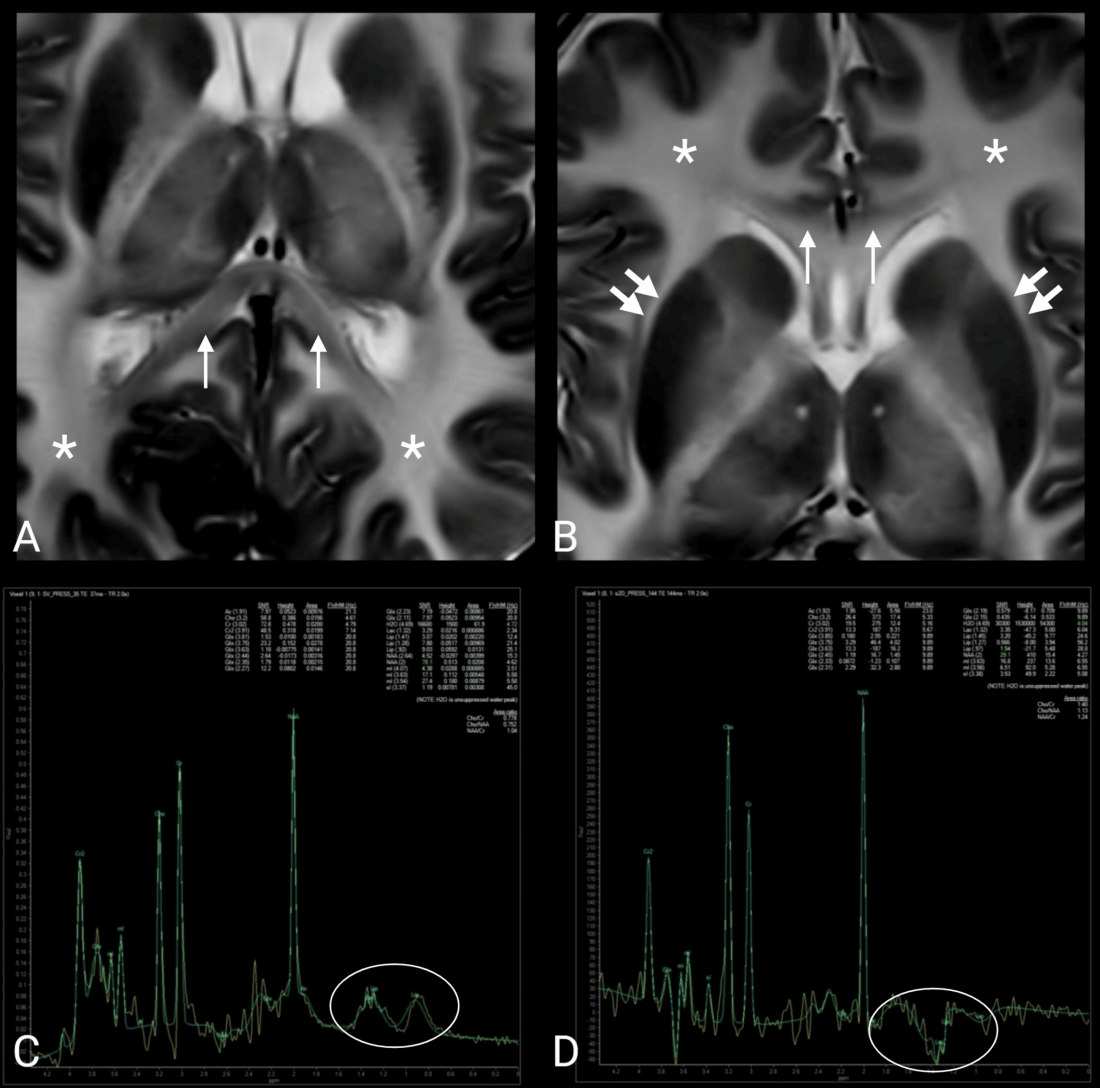
Relative sparing of the corpus callosum and lactate on MR spectroscopy. (A, B) Axial T2-weighted images at the level of the thalami demonstrate diffuse supratentorial white matter (white asterisks) T2 hyperintensity with relative sparing of a subset of callosal fibers (white arrows), most evident in the splenium (comparatively lower T2 signal than the remainder of the supratentorial white matter). No definite basal ganglia (double white arrows) signal abnormality is identified. (C, D) Single-voxel proton MR spectroscopy (short and intermediate echo times) acquired at the level of the basal ganglia demonstrates an inverted lactate doublet (white ovals); the remaining metabolite profile is nonspecific.

The internal capsules and corpus callosum were extensively involved. Relative sparing of a subset of callosal fibers, most notably within the splenium, was suggested by a comparatively lower T2 signal intensity than in the remainder of the supratentorial white matter (Figure 4). No definite signal abnormalities were identified in the basal ganglia or cerebral cortex.

Single-voxel proton MR spectroscopy (short and intermediate echo times) was performed at the level of the left basal ganglia (voxel size 2 × 2 × 2 cm) and demonstrated an inverted lactate doublet. The remaining metabolite profiles were nonspecific and did not suggest a characteristic diagnostic pattern (Figure 4).

Timeline summary

The patient exhibited early developmental delay before the acute event, characterized by achieving head control at six months and an inability to sit independently within the context of parental consanguinity. At six months, the patient experienced febrile rotavirus gastroenteritis, with a fever reaching 38.5°C, accompanied by vomiting and diarrhea, which preceded the onset of seizures on the third day. Initially presenting as focal motor seizures, these episodes rapidly increased in frequency to multiple occurrences per day; administration of levetiracetam reduced but did not eliminate the seizures. In the following weeks, the infant experienced immediate motor regression, losing head control and the ability to sit, along with diminished purposeful hand use. The seizures progressed to generalized tonic-clonic seizures with clusters of myoclonic jerks. Neuroimaging at this stage revealed diffuse symmetric supratentorial white matter T1 hypointensity/T2 hyperintensity without U-fiber sparing, advanced white matter rarefaction (deep white matter FLAIR hypointensity), and superimposed multifocal diffusion restriction affecting areas including the internal capsules, corpus callosum, mammillothalamic tracts, optic radiations, and dentate nuclei. MR spectroscopy (left basal ganglia single voxel) indicated a lactate doublet. Metabolic evaluation yielded unremarkable results, and a mitochondrial cocktail with levetiracetam was administered for presumed mitochondrial encephalopathy, resulting in only transient improvement. The clinical course remained progressive, with ongoing daily seizures, including two ICU admissions for status epilepticus at seven and eight months; valproate was introduced, but seizure frequency increased. At eight months, neurological examination revealed marked axial hypotonia with brisk reflexes, evolving lower limb spasticity, and severe loss of motor milestones, while visual tracking and auditory responses were preserved. Subsequent administration of clobazam, lacosamide, and valproate reduced the seizure burden without achieving complete control. The infant continued to deteriorate and succumbed at approximately nine months of age, approximately three months after clinical onset. A summary of the clinical case is presented in Table 1.

**Table 1:** Summary timeline of key clinical, imaging, and genetic events. Summary timeline of key clinical, radiologic, laboratory, findings, treatments, and outcome in an infant with severe *EIF2B5*-related vanishing white matter disease, organized chronologically to facilitate rapid clinical cross-reference. CBC = complete blood count; FLAIR = fluid-attenuated inversion recovery; MRS = MR spectroscopy

Timepoint	Key clinical features	Seizure semiology	Imaging	Laboratory tests	Management and outcome
Background	Term male infant; first-cousin parents; early developmental delay (head control achieved at six months; not sitting independently)	—	N/A	N/A	—
6 months (trigger)	Febrile rotavirus gastroenteritis (fever up to 38.5°C, vomiting, diarrhea)	On the third day of illness, the patient began experiencing focal motor seizures, which subsequently progressed to multiple episodes per day	N/A	N/A	Levotiracetam reduced but did not abolish seizures
Post-infectious period (weeks after onset)	Immediate developmental regression: loss of head control and sitting ability; reduced purposeful hand use; continued decline without new gains	Generalized tonic-clonic seizures and clusters of myoclonic jerks. The infant was awake and alert between seizures; ictal awareness appeared variably impaired	MRI: diffuse symmetric supratentorial white matter T1 hypointensity/T2 hyperintensity without U-fiber sparing; multifocal diffusion restriction (internal capsules, corpus callosum, mammillothalamic tracts, optic radiations, dentate nuclei); deep white matter FLAIR hypointensity consistent with advanced rarefaction. MRS: left basal ganglia single voxel—lactate doublet	Metabolic evaluation unremarkable (lactate, ammonia, plasma amino acids, urine organic acids, acylcarnitine profile); CBC and liver function tests within reference ranges	“Mitochondrial cocktail” + levetiracetam given for presumed mitochondrial encephalopathy with transient improvement
7–8 months	Ongoing daily seizures; two ICU admissions (at 7 and 8 months) for status epilepticus; no invasive ventilatory support beyond oxygen	Generalized tonic-clonic seizures and clusters of myoclonic jerks	N/A	N/A	Sodium valproate. Despite medication, seizure frequency increased
8 months	Marked axial hypotonia with brisk deep tendon reflexes; no head control, unable to sit, no purposeful hand use; preserved visual tracking and auditory responses; lower-limb spasticity noted	Generalized tonic-clonic seizures and clusters of myoclonic jerks	N/A	N/A	Clobazam + lacosamide + sodium valproate further reduced seizure burden without complete control
9 months	Progressive course with ongoing deterioration	Generalized tonic-clonic seizures and clusters of myoclonic jerks	N/A	N/A	Death ~3 months after clinical onset

Genetic findings

Given the parental consanguinity and the clinical-radiological phenotype, molecular testing was pursued. A targeted leukodystrophy gene panel (whole-exome sequencing) identified a homozygous missense variant in *EIF2B5* (NM_003907.3:c.584G>A), predicted to result in p.(Arg195His). This variant has been previously reported in association with VWM disease and is classified as pathogenic. No other pathogenic or likely pathogenic variants were detected in the remaining genes included in the leukodystrophy panel. Identification of homozygous *EIF2B5* p.(Arg195His), a variant previously reported in severe/early-onset VWM, established the molecular diagnosis.

Parental segregation testing was not performed; however, the first-cousin consanguinity and the patient’s homozygosity for *EIF2B5* NM_003907.3:c.584G>A (p.Arg195His) were consistent with autosomal recessive inheritance, and parental heterozygous carrier status was likely.

## Discussion

This infant’s clinical course with stressor-associated developmental regression in the second half of the first year of life, rapid motor deterioration with axial hypotonia and pyramidal signs/spasticity, and refractory seizures is consistent with subacute infantile-onset VWM disease. In VWM, neurological decline is typically progressive but is characteristically punctuated by episodes of rapid deterioration triggered by physiological stressors, most commonly febrile infections, as observed in the present case [1,2].

The entity formerly designated CLE, described in the Cree population of Canada, is now recognized as an early infantile-onset form of VWM disease [5]. Molecular studies have established that CLE is allelic with VWM at the *EIF2B5* locus. Fogli et al. showed that affected Cree infants were homozygous for the *EIF2B5* c.584G>A (p.Arg195His) variant, whereas unaffected relatives were either heterozygous carriers or wild type, supporting allelic identity between CLE and VWM [5].

The history of initial milestone acquisition followed by rapid loss of skills after an intercurrent illness aligns with the typical VWM trajectory, in which early development may be normal or only mildly delayed before stressor-related decompensation and subsequent decline. Because infants cannot manifest gait ataxia, cerebellar involvement may be clinically difficult to demonstrate; nevertheless, the combination of profound axial hypotonia and evolving spasticity is compatible with the recognized cerebello-spastic phenotype of VWM. Preserved visual tracking and hearing are also commonly maintained early in the disease course.

Epilepsy is frequent in VWM, particularly in infantile-onset disease [1,2,6]. In a large multicenter natural-history cohort, seizures occurred in approximately 60% of patients, with a lower incidence in later-onset groups. Seizure semiology was heterogeneous, including non-motor and focal-onset seizures with and without impaired awareness; generalized onset tonic-clonic seizures were reported as the most common type [7].

*EIF2B5* mutations have been reported across a broad age-at-onset spectrum, from prenatal presentations to adulthood, with earlier onset generally correlating with more severe disease [8]. In a cohort of 13 patients, most individuals demonstrated a childhood-onset phenotype, whereas two presented in adulthood and one exhibited late-infantile onset [9]. MRI typically demonstrates bilateral, symmetrical T2 hyperintensity of the deep and periventricular white matter, with subcortical U-fiber involvement, cystic degeneration predominantly in the frontotemporal regions, and corpus callosum abnormalities. A characteristic “stripe-like” white matter pattern, attributed to relative perivascular sparing, has also been described in *EIF2B5*-related VWM, although it was not observed in the present case. Additional reported findings include globus pallidus and thalamic involvement, occasional optic nerve atrophy, and absence of abnormal contrast enhancement. Cerebellar white matter and dentate nucleus involvement are frequently observed. The homozygous *EIF2B5* p.(Arg195His) variant is currently classified as pathogenic for VWM disease, and GeneReviews describes it as a “Cree founder variant” associated with a particularly severe clinical phenotype [8].

Dooves et al. (2016) generated mouse models carrying the *Eif2b5* p.(Arg191His) mutation, the murine equivalent of the human p.(Arg195His) variant [10]. They reported impaired maturation of white matter astrocytes preceding overt clinical disease, underscoring astrocytes as key contributors to the underlying pathomechanism. This missense change affects the ε-subunit of eIF2B and has been associated with marked impairment of eIF2B function, consistent with a severe phenotype. The MRI appearance in our patient closely parallels that reported in CLE, and is highly concordant with the imaging pattern described by Alorainy et al [11].

Deng et al. provided genotype-stratified age-at-onset data from which we identified six individuals homozygous for *EIF2B5* p.(Arg195His), with reported onset ages ranging from 0.3 to 0.9 years. The median age at onset for this subgroup was 0.65 years, calculated as the mean of the two central values (0.6 and 0.7 years; n = 6). These findings corroborate the classification of p.(Arg195His) as a severe early-infantile genotype, consistent with the phenotype observed in our case.

VWM is inherited in an autosomal recessive manner; in this case, it is attributable to biallelic pathogenic variants in *EIF2B5*. Within this framework, individuals in whom one allele is p.(Arg195His), typically in trans with a second pathogenic *EIF2B5* variant (compound heterozygosity), have been reported to present in early infancy, although age at onset is variable [12]. In contrast, homozygosity for p.(Arg195His) appears more consistently associated with infantile and severe disease. Adult-onset presentations have also been described in compound heterozygous genotypes that include p.(Arg195His), underscoring the phenotypic variability observed across biallelic *EIF2B5* variant combinations [13].

The eIF2B decamer comprises a regulatory subcomplex (α/β/δ) and catalytic subunits (γ/ε), with the ε-subunit providing the guanine nucleotide exchange activity that converts eIF2-GDP to its active GTP-bound form, thereby enabling initiation of mRNA translation [14]. Stressors such as infection induce phosphorylation of eIF2α (eIF2α-P); eIF2α-P then binds eIF2B and inhibits its GEF activity. Under physiological conditions, this response transiently attenuates global protein synthesis while activating the integrated stress response (ISR), thereby promoting cellular adaptation. In the setting of EIF2Bε dysfunction with reduced catalytic activity, however, this stress response becomes maladaptive: impaired eIF2B activity leads to prolonged suppression of translation, compromising oligodendrocyte and astrocyte homeostasis and ultimately contributing to progressive white matter rarefaction. This mechanism offers a biological rationale for the characteristic rapid clinical deterioration precipitated by fever or trauma in VWM.

Preventive measures are central to the management of VWM. Our patient’s course underscores this principle: marked deterioration following a febrile rotavirus illness mirrors the characteristic stressor-related exacerbations described in VWM. At present, no disease-modifying therapy is available, and management remains supportive. Anti-spasticity treatment and physical rehabilitation may mitigate motor impairment, while antiseizure medications are used for seizure control. Genetic counseling is essential. As an autosomal recessive disorder, the recurrence risk is 25% for each pregnancy when both parents are carriers, and the family was counseled accordingly.

## Conclusions

This case study exemplifies a severe phenotype associated with *EIF2B5*-related VWM, characterized by infection-induced neurological deterioration, significant motor regression, and, ultimately, a fatal outcome. While acknowledging the inherent limitations of single-patient reports, these findings should be considered as supportive rather than widely generalizable. Nevertheless, the documentation of the *EIF2B5* p.(Arg195His) mutation in an Azerbaijani family broadens the geographic and ethnic context of this severe genotype, which has been predominantly reported in the Cree founder population. This underscores the importance of inclusive genetic testing for leukodystrophies, including *EIF2B* genes, in underrepresented populations. Early molecular confirmation can minimize unnecessary diagnostic procedures and facilitate timely prognostic and genetic counseling. Moreover, preventive supportive care, particularly the early and aggressive management of febrile illnesses and other stressors, remains central to the management of VWM.
